# Mechanical stretching boosts expansion and regeneration of intestinal organoids through fueling stem cell self-renewal

**DOI:** 10.1186/s13619-022-00137-4

**Published:** 2022-11-02

**Authors:** Fanlu Meng, Congcong Shen, Li Yang, Chao Ni, Jianyong Huang, Kaijun Lin, Zanxia Cao, Shicai Xu, Wanling Cui, Xiaoxin Wang, Bailing Zhou, Chunyang Xiong, Jihua Wang, Bing Zhao

**Affiliations:** 1 Shandong Key Laboratory of Biophysics, Institute of Biophysics, Dezhou University, Dezhou, 253023 China; 2 Department of Mechanics and Engineering Science, College of Engineering, Peking University, Beijing, 100871 China; 3 State Key Laboratory of Genetic Engineering, School of Life Sciences, Zhongshan Hospital, Fudan University, Shanghai, 200438 China; 4 Beijing Innovation Center for Engineering Science and Advanced Technology, College of Engineering, Peking University, Beijing, 100871 China; 5 Wenzhou Institute, University of Chinese Academy of Sciences, Oujiang Laboratory, Wenzhou, 325000 Zhejiang China

**Keywords:** Mechanical stretching, Intestinal organoid, Lgr5^+^ stem cell, Regeneration, Wnt/β-catenin signaling

## Abstract

Intestinal organoids, derived from intestinal stem cell self-organization, recapitulate the tissue structures and behaviors of the intestinal epithelium, which hold great potential for the study of developmental biology, disease modeling, and regenerative medicine. The intestinal epithelium is exposed to dynamic mechanical forces which exert profound effects on gut development. However, the conventional intestinal organoid culture system neglects the key role of mechanical microenvironments but relies solely on biological factors. Here, we show that adding cyclic stretch to intestinal organoid cultures remarkably up-regulates the signature gene expression and proliferation of intestinal stem cells. Furthermore, mechanical stretching stimulates the expansion of SOX9^+^ progenitors by activating the Wnt/β-Catenin signaling. These data demonstrate that the incorporation of mechanical stretch boosts the stemness of intestinal stem cells, thus benefiting organoid growth. Our findings have provided a way to optimize an organoid generation system through understanding cross-talk between biological and mechanical factors, paving the way for the application of mechanical forces in organoid-based models.

**Supplementary Information:**

The online version contains supplementary material available at 10.1186/s13619-022-00137-4.

## Background

The intestinal organoid culture system was first established in 2009 by Hans Clevers’ laboratory by employing the self-organization ability of Lgr5^+^ stem cells (Sato et al. 2009). This pioneering work has adopted a biological and mechanically static methodology in creating a three-dimensional (3D) ‘crypt-villus’ structure that contains multiple differentiated cell types found in the intestinal epithelium (Barker 2014). This culture system requires two essential components: one is to embed cells within matrigel to support 3D organoid structures, and the other is to perturb stemness-related signaling pathways by supplementing the culture medium with growth factors including EGF (epidermal growth factor), R-Spondin (the Lgr5 ligand and Wnt agonist), and Noggin (BMP inhibitor). As intestinal organoids can recapitulate vivo-like structural and functional characteristics, they are widely applied to investigate intestinal morphogenesis and disease pathogenesis and have been used as potential diagnostic and therapeutic tools over the last few decades (Fritsche et al. 2021; Geurts et al. 2021; Kim et al. 2020; Lau et al. 2020; Rahmani et al. 2019; Ramani et al. 2018; Sato and Clevers 2013).

The intestinal epithelium is organized into crypts-villi units. It is the most rapidly self-renewing tissue. Lgr5+ stem cells localized at the base of crypts produce the proliferating progenitors and transit-amplifying (TA) cells, driving the renewal process (Barker et al. 2007). TA cells migrate away from the crypt base, differentiate into the postmitotic lineages at the villus tips, then undergo apoptosis and slough into the lumen of the intestine (Lehrer et al. 1998). This vigorous cell proliferation process ensures a rapid epithelial cell turnover within 4–5 days to retain the overall epithelium homeostasis (Barker et al. 2010; van der Flier and Clevers 2009). Epithelial cells are subjected to a myriad of physical forces in the intestinal environment, including the pressure and shear from endoluminal chyme, the cyclic strain associated with rhythmic villous motility, and intestine peristalsis induced cyclic deformation caused by muscular contraction and relaxation deeper within the bowel wall (Basson 2003; Gayer and Basson 2009). The peristalsis contractions of the small bowel occur at frequencies of 7–20 per minute (Grivel and Ruckebusch 1972; Otterson and Sarr 1993). Proliferation of intestinal epithelial cells in response to mechanical strain is validated by large amounts of studies. Increasing stretch in vivo in pig small intestines drives cell division and proliferation (Spencer et al. 2006), and these proliferative effects are also observed in immortalized cell lines (Blair et al. 2015; Zhang et al. 2006; Zhang et al. 2003). Native intestinal epithelium retains a dynamic proliferating and regenerating state under a mechanically induced complex physical condition.

Despite the resemblance of intestinal organoid to the epithelial tissue of the native intestine in many aspects, such as maintaining the functional intestinal lineages (e.g., Paneth cells, goblet cells, enteroendocrine cells, enterocytes, and tuft cells) and “villus-crypt” architecture (Haber et al. 2017; Sato and Clevers 2013; Sato et al. 2009), the neglection of mechanical effects for conventional static organoid culture system limits its utility in many ways. Recently, there has been renewed interest in not only the biological cues in organoid studies, but also the mechanics of organoid formation, expansion, and regeneration (Buske et al. 2012; Kwon et al. 2020; Li et al. 2021; Perez-Gonzalez et al. 2021b). Several studies are focused on intrinsic mechanics. With intestinal organoid as a typical model, the mechanism of symmetry-breaking (Serra et al. 2019), crypt formation (Tallapragada et al. 2021; Yang et al. 2021) and cell migration (Perez-Gonzalez et al. 2021a) is intensively investigated, indicating the mechanical influences during organoid development and morphogenesis. For studies on extrinsic mechanical cues, novel synthetic ECM (Extracellular Matrix) (Bergenheim et al. 2020; Broguiere et al. 2018; Brown and Mills 2017; Cruz-Acuna et al. 2017; Gjorevski and Lutolf 2017; Gjorevski et al. 2016; Hernandez-Gordillo et al. 2020; Ng et al. 2019; Schlieve and Grikscheit 2020; Tong et al. 2018) and biomimetic-based scaffolds (Gjorevski et al. 2022; Roh et al. 2019; Wang et al. 2017), are introduced to boost organoid culture. Moreover, in a recent work concerning the impact of mechanical forces on the growth and maturation of human intestinal organoid (HIO), Polling et al. transplanted HIOs into the mesentery of NOD-SCID mice with a nitinol spring for the purpose of incorporating mechanical strain with HIO generation (Poling et al. 2018). Harvested grafts were identified to maintain increased intestinal and maturation features in comparison with those unstretched organoid grafts. Progresses in organoid mechanobiology are indeed remarkable, but knowledge on cross-talk between the biotic and abiotic factors is still limited in essence, and some of the mentioned studies on combining mechanical cues with intestinal organoid culture system are, to some extent, laborious and technically difficult to perform in laboratories, thus limiting their utility. How mechanical forces regulate the physiological growth condition of the multicellular homeostasis and how to integrate mechanical cues to promote organoid culture efficiency are still not clear.

Here, we developed a mechanically dynamic method based on cyclic stretching for intestinal organoid culture. In contrast to static conditions used in the traditional organoid culture, mechanical stretching with refined parameter settings remarkably promoted intestinal organoid stemness, including up-regulation of stem cell marker genes and proliferation of stem cells. Meanwhile, it appeared that Wnt/β-Catenin, which is an essential signaling pathway in the maintenance of stemness, was activated by mechanical stretching. This method for manipulating mechanical cues within the organoid culture system provides an ideal research model for exploring the cross-talk between mechanical cues and biological factors, which deepens our understanding of mechanical microenvironments that drives organoid regeneration and simultaneously broadens the application of stretching-induced organoid culture in regenerative medicine.

## Results

### Integration of cyclic stretch into the intestinal organoid culture

**Fig. 1:**
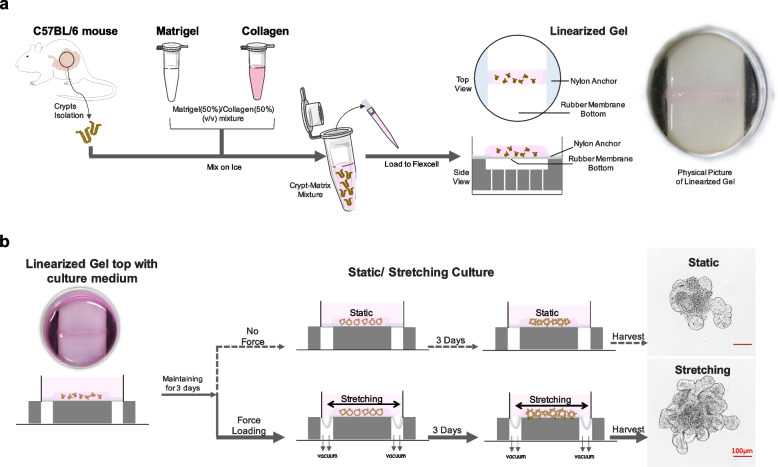
Schematics of mechanical stretching induced organoid culture system. **a** Crypts were isolated and mixed with matrix to create gel strip. **b** Linearized gel wrapped with crypts was sustained for three-day static incubation, then subjected to stretching for three days, and finally harvested on day 7 for imaging, data acquisition, and analysis, or to passage for another round of stretch. Organoids incubated under static conditions during the whole process were selected as a control group. Stretching-induced organoid exhibited size and crypt number increase in comparison with the static culture. Scale bar, 100 μm

The intestinal organoid culture system was initially established by employing stem cell self-organizing abilities and perturbating signaling pathways through a temporal series of growth factor manipulations. Although this exclusively biological and mechanically static methodology has succeeded in creating functional intestinal lineages and architecture similar to those of the native intestine, the role of mechanical cues in systems dominated by biological factors has not been addressed. To recapitulate the mechanical cues in the intestinal organoid culture system, we have developed a mechanically dynamic culture method through combing cyclic stretch with the intestinal organoid generation process, which significantly induces an increase in external size, crypt number, and stem cell proliferation of intestinal organoids (Fig. 1).

Mouse intestinal crypts used as an initiation of Mouse Intestinal Organoid (MIO) culture were isolated as described. Obtained crypts mixed evenly with appropriate matrix were loaded to Flexcell Culture System to create a linear-shaped 3D construct by depositing rubber-based culture plate and trough loader under vacuum conditions (Fig. 1a and Supplementary Video 1). Solidified gel strip was topped with a 2.5 ml crypt culture medium, sustained for three-day incubation, and then exposed to cyclic stretch for mechanically induced dynamic cultivation for 3 days (Fig. 1b and Supplementary Videos 2 and 3). Organoids were harvested on day 7 for imaging, data acquisition, and analysis, or passaging for another round of culture. To get the matrix supporting organoid growth adapted to the mechanically dynamic changed culture system, we optimized the most commonly used Matrigel (Corning) by adding type I collagen (Thermo) to create Matrigel (50%)/collagen (50%) (v/v) mixture, which could both afford the organoid survival and adapt to the stretching system (Fig. S1). We found that stretched organoids (8% cyclic strain) displayed increased organoid size and crypt number in comparison to control organoids under static culture conditions (Fig. 1b). To gain a deeper understanding of the cyclic stretching stimulated mechanical effects, we next collected organoid generated under stretching or static conditions, and performed further functional dissection at the cellular and molecular levels.

### Cyclic stretch induces organoid growth and regeneration in a strain-dependent manner

**Fig. 2:**
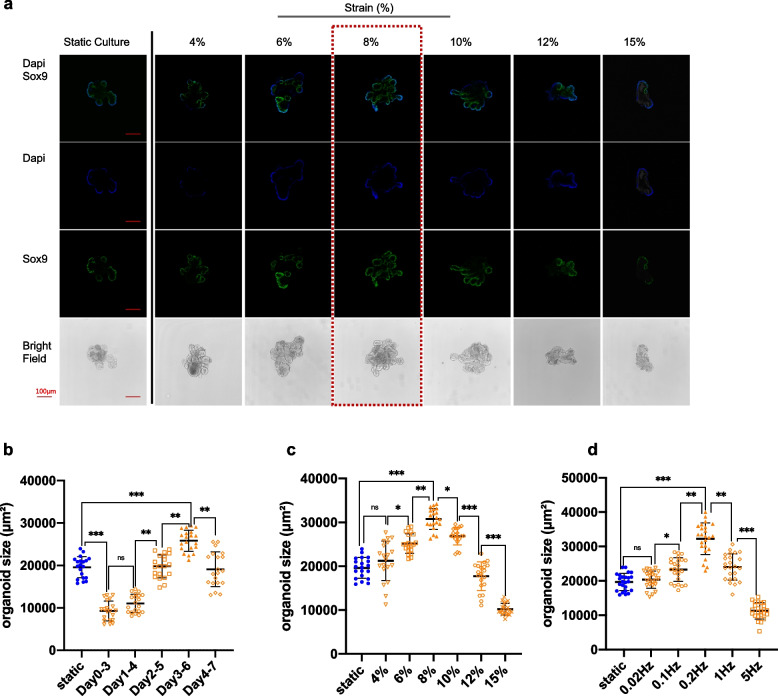
The effect of strain magnitude, frequency, and timing window of mechanical force on organoid culture. **a** Imaging of cyclic stretch (0.2 Hz) started on Day 3 at various amplitudes, with 8% strain to be an optimum condition. **b**, **c** and **d** showed the data analysis for strain timing, magnitude, and frequency. The statistical data represent mean ± s.d. (*n* = 6 organoids derived from three independent experiments for each condition). Student’s t-test: ***P < 0.001. **P < 0.01. *P < 0.05. Scale bar, 100 μm

The effects of frequency, magnitude, and stretch-loading timing window were investigated. In the first set of experiments, 10% strain at the frequency of 0.2 Hz was separately applied on days 0, 1, 2, 3, and 4 after crypt isolation, stretched for 3 days, and uniformly harvested on day 7. Day 3–6 turned out to be a desirable timing window for stretching, as the organoids exhibited larger size and elevated stemness than those under the static culture condition. Too early mechanical force loading hampered organoid expansion, while the delayed introduction of stretching stimulation did not cause many phenotypic differences between the stretching and static groups (Fig. 2b and Fig. S2a).

We next examined how the strain amplitude affects organoid growth. The same ‘Day 3–6’ loading time point was applied at the frequency of 0.2 Hz. Intestinal organoids under the condition of 8% cyclic stretching exhibited larger size and higher stemness than those in the static control group. The excessive strain may have created very high stresses on the cytoskeleton that would cause it to retract, leading negative effect on MIO growth, while mechanical strain with amplitude under 10% stimulated MIO expanding with the optimum at 8% (Fig. 2a and c). These results demonstrated the pivotal role of strain amplitude in controlling organoid growth.

Moreover, 8% cyclic stretch at the frequency in the range of 0.02–5 Hz resulted in an increase in organoid expansion, and immunostaining for SOX9 indicated that stretched organoids contained an enriched population of stem cells compared to static cultures. The optimal frequency was identified as 0.2 Hz, which approximates the physiological peristaltic frequency of the small intestine. However, high stretching frequency hampered organoid formation instead of boosting growth (Fig. 2d and Fig. S2b).

Accordingly, this stretching condition of 8% strain with Day3–6 duration at the frequency of 0.2 Hz (12 cycles/min) was determined to be the optimal condition for inducing organoid growth and regeneration (Fig. 2 and Fig. S2). In summary, results suggested that strain, timing window, and cyclic frequency cooperatively regulate the influence of mechanical stretching effect on organoid culture.

### Cyclic stretch induces crypt hyperplasia in organoids

**Fig. 3:**
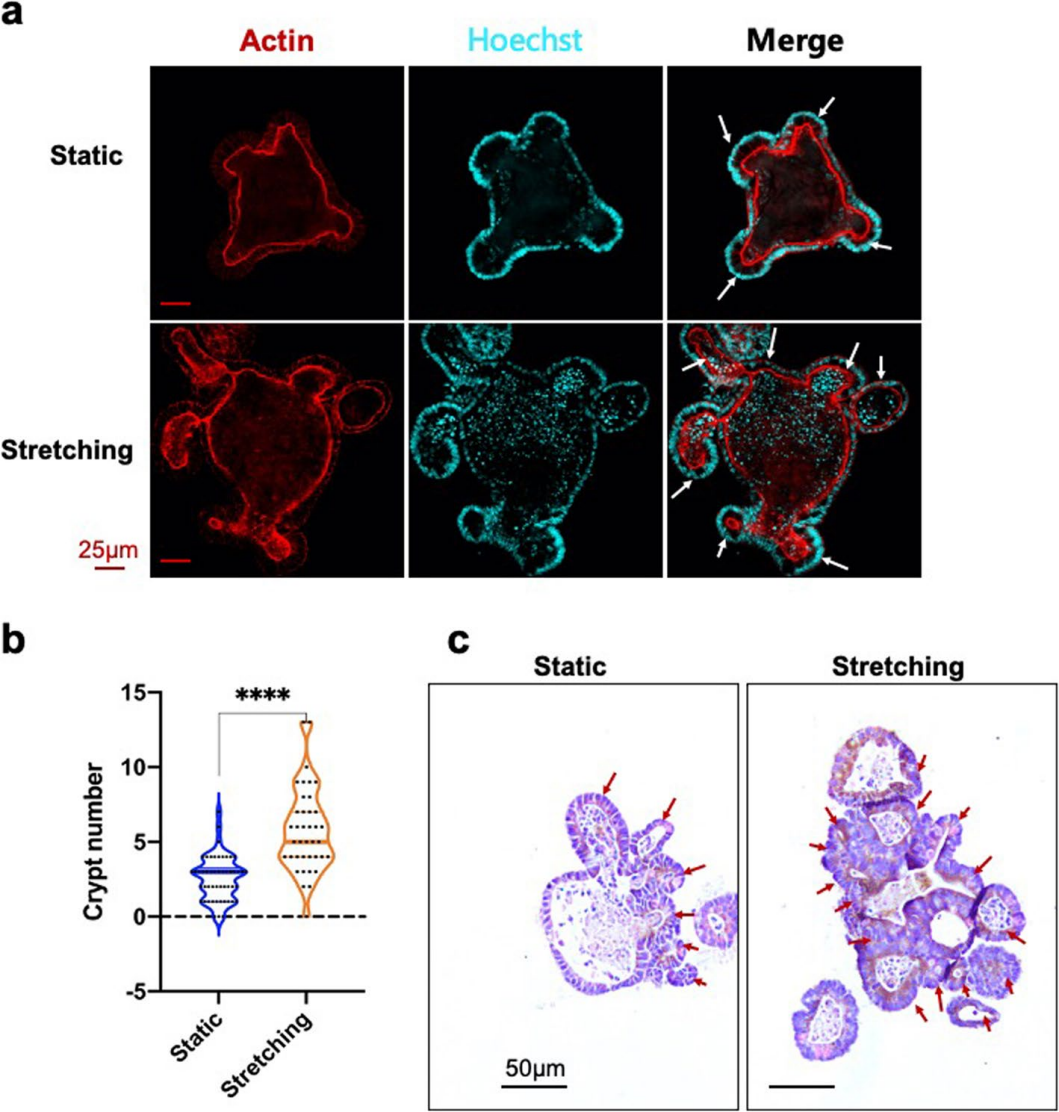
Stretching induced crypt hyperplasia. **a** Immunostaining for β-actin. **b** Statistical analysis for the number of crypt fission. **c** Haematoxylin and Eosin-staining. The statistical data represent mean ± s.d. (*n* = 30 organoids derived from three independent experiments for each condition). Student’s t-test: ***P < 0.001. **P < 0.01. *P < 0.05. Scale bar, 25 μm in **a** and 50 μm in **c**

Intestinal crypts are the hub of Paneth cells and intestinal stem cells (ISCs), which fuel the active self-renewal of the epithelium. Crypt hyperplasia is associated with increased epithelial proliferation and may involve ISC expansion and crypt fission. Crypt hyperplasia frequently occurs in organoids, so it can be used as an indicator to evaluate organoid growth and morphogenesis. Immunostaining for β-actin presented the clear skeleton architecture of intestinal organoids. The confocal imaging revealed phenotypic differences between stretched and unstretched organoids (Fig. 3a). In contrast to organoids maintained under static conditions, those exposed to mechanical stretch showed a more spread budding-like morphology, which in turn contributed to the organoid expansion. To further validate this finding, we quantified architectural features of the organoid of passage 3 using paraffin sections and haematoxylin and eosin (H&E) staining. H&E staining indicated that the crypt number per organoid section for stretching was more than double that of organoids in static culture (Fig. 3b and c), which was consistent with the corresponding β-actin immunostaining. These data collectively demonstrated the key role of mechanical stretch in promoting crypt formation and hyperplasia, enhancing architectural complexity, and enlarging the size of organoids.

### Cyclic stretch manipulates cell proliferation patterns in intestinal organoids

**Fig. 4:**
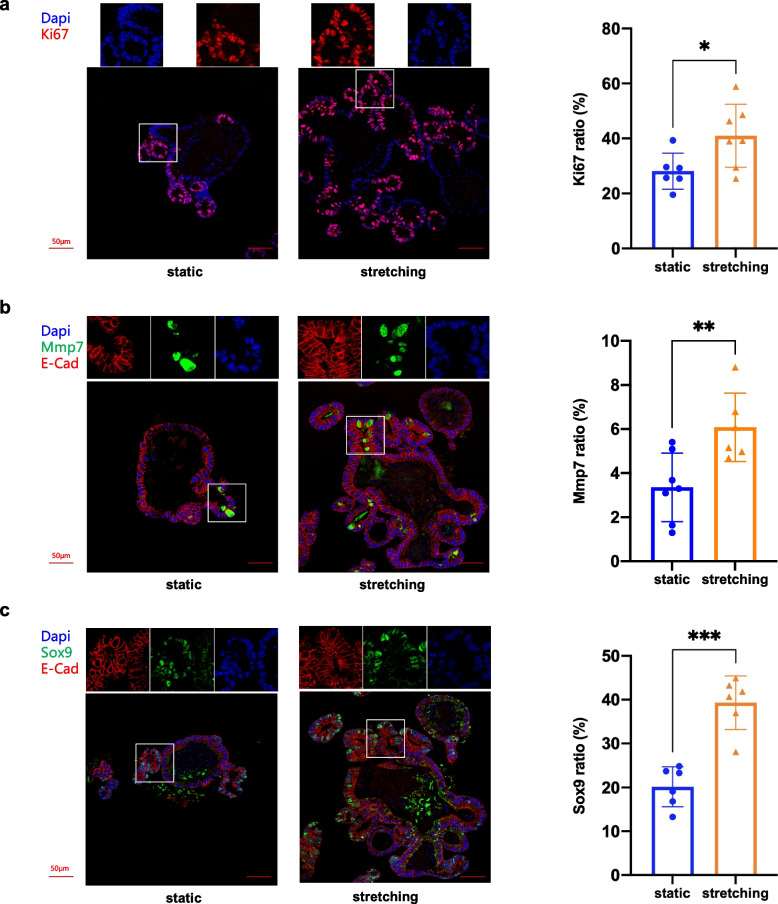
Mechanical stretching induces cell proliferation. **a** Imaging and quantification of IF staining for Ki67 in organoid cultured with 8% cyclic stretching (right panel) or under static condition (left panel), respectively. **b** Imaging and quantification of IF staining for Mmp7 in organoid cultured with 8% cyclic stretching (right panel) or under static condition (left panel), respectively. **c** Imaging and quantification of IF staining for Sox9 in organoid cultured with 8% cyclic stretching (right panel) or under static condition (left panel) respectively. *n* = 6 organoids derived from three independent experiments for each condition. Student’s t-test: ***P < 0.001. **P < 0.01. *P < 0.05. Scale bars, 50 μm

To detect changes in cellular levels in stretched organoids, we analyzed paraffin sections of organoid samples with immunohistochemical (IHC) staining and immunofluorescence (IF) assays. Different cell markers were chosen for the purpose of quantification of different cell types. IHC staining for proliferative marker Ki67 (MKi67) was performed to examine the proliferative capacity of the cells. Staining showed that the percentage of Ki67^+^ cells for stretched organoids was ~ 75%, which was higher than that seen in control organoids (~ 60%), indicating that stretched organoids had increased proliferative capacities (Fig. S3a). Immunofluorescence staining for Ki67 also revealed a notable difference (stretching ~ 40%, static ~ 23%) (Fig. 4a), which was consistent with the stretching-induced upward trend exhibited by IHC, although the actual ratios originating from these two detecting methods were slightly different due to their discrepancy in sensitivity and accuracy.

Immunostaining was also performed for Mmp7, the marker of Paneth cells which are mainly localized within crypts, reside between Lgr5 stem cells, and constitute the niche for ISCs. Results of Immunofluorescence suggested that the Mmp7^+^ cell ratio of the stretching group (~ 6%) was almost twice that of the control group (~ 3%) (Fig. 4b). IHC staining also showed an increase in Mmp7^+^ cell ratio in stretched organoids (~ 7%) compared with that of unstretched groups (~ 5%) (Fig. S3b).

Staining for stem cell marker Sox9 showed a vast variation between stretched and static culture. Immunofluorescence for Sox9 exhibited a significant increase in Sox9^+^ cells ratio (40.8%) induced by mechanical stretch, which was almost twice that of the organoids generated under static condition (20.2%) (Fig. 4c). IHC staining indicated that Sox9^+^ cells accounted for 47.7% in static culture and 70.5% in mechanical stretching condition, which was in good agreement with results of immunofluorescence (Fig. S3c). Regarding our study, cyclic stretching induced the expansion of intestinal organoids. Vigorous cell proliferation, especially stem cell proliferation, was observed within the stretched organoid culture (Fig. 4, Fig. S5b and S6). We speculate that it may be owing to the stem cell activation in response to mechanical stretching that fuels cell proliferation, thus boosting the robust growth of the organoid culture.

### Cyclic stretch enhances stemness in intestinal organoids

**Fig. 5:**
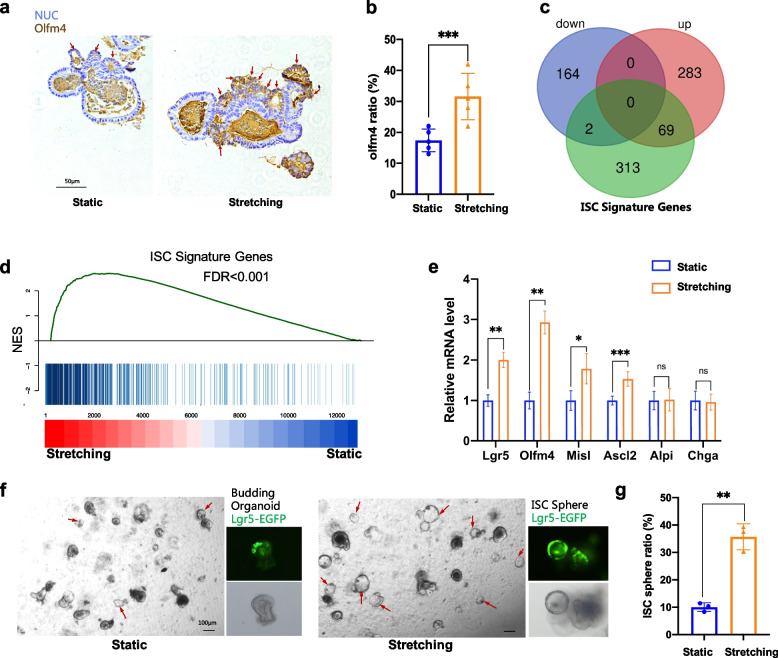
Stretching induced stemness promotion. **a** and **b** showed the imaging and quantification of IHC staining for Olfm4. **c** Venn diagram showing the overlap among ISC signature genes (green), up-regulated genes (red), and down-regulated genes (purple). The significance was evaluated by Fisher’s exact test. **d** GSEA plot of static and stretching treated organoid sample compared with a gene list containing the top 384 ISC signature genes. NES, normalized enrichment score. **e** Quantification of the expression level of Lgr5, Ascl2, Olfm4, Msi1, Alpi, and Chga using qRT-PCR. **f** Images of organoids expressing Lgr5-eGFP at day1 post passaging. The right images were for stretching and the left for static. Red arrows marked out the round transparent ISC sphere complexes. **g** Quantification of ISC sphere ratio. Student’s t-test: ***P < 0.001. **P < 0.01. *P < 0.05. Scale bars, 50 μm in** a**, 100 μm in** f**

A series of experiments were performed to test stemness-relevant gene expression profiles. Olfm4 was a typical stemness marker gene, which was chosen to indicate the regeneration ability of organoids. The results of IHC staining for Olfm4 indicated that the stained positive cell counterparts showed a higher ratio in stretched organoid compared with static culture (Fig. 5a, b, and Fig. S4).

RNA sequencing (RNA-Seq) was performed to develop a global view of differential gene expression between stretching and static culture. Out of 12,633 genes annotated in the genome, 518 were significantly differentially regulated among the samples, including 166 down-regulated and 352 up-regulated (Fig. 5c and Fig. S5a). A Venn diagram was applied to show the overlap between differentially expressed genes (DEGs) and ISC signature genes (Fig. 5c, Table S1). The up-regulated gene set and ISC signature gene set shared 69 genes (Fig. 5c, Table S2), including stem cell marker genes, such as Lgr5, Olfm4, Ascl2, etc. Gene set enrichment analysis (GSEA) showed highly significant enrichment (false discovery rate (FDR) and *P*-value, 0.0001) of the ISC signature gene set towards the upregulated genes after stretching (Fig. 5d). We further verified the up-expression of several crucial stemness marker genes using real-time quantitative PCR. Results suggested that, in comparison with the static culture condition, stretching induced Lgr5, Olfm4, Msil, and Ascl2 to express at higher levels. Nevertheless, not much difference was observed for markers of enterocytes (Alpi) as well as entero-endocrine cells (Chga) between the stretched and control (Fig. 5e).

ISC spheres maintained transparent and round morphology with elevated stemness due to the high purity of stem cells within the sphere structure. As a result, an increased ISC ratio could be considered a marker of elevated stemness. Interestingly, an increased portion of organoids exhibited a typically globular phenotype with high-purity stem cells during progressive in vivo passages (Fig. 5f). ISC sphere ratio of stretched MIO was~ 35% for P3, which was much higher than ~ 10% for control (Fig. 5g), indicating enhanced stemness driven by continuous stretching. Overall, mechanical stretching greatly enhanced the stemness of organoid culture, including up-regulating the transcription and expression of stemness-relevant genes and increasing the ISC sphere ratio when performing organoid passaging.

### Cyclic stretch activates the Wnt/β-catenin signaling pathways to sustain a high stemness level

In response to stretching stimulation, ISC signature gene expression and stem cell proliferation were remarkably boosted as indicated by immunostaining and gene sequencing analysis (Figs. 4 and 5). ISCs self-renew under a high level of Wnt/β-catenin signaling and go through differentiation when the Wnt/β-catenin signaling subsides in the intestinal crypt. To this end, we speculated that cyclic stretching could activate the Wnt/β-Catenin pathway, which was highly relevant to intestinal stemness maintenance. To test this hypothesis, we removed growth factor R-Spondin1 from the crypt cultural medium on Day 3 just before the mechanical force loading point and then started stretching as described before (Fig. 1).

**Fig. 6:**
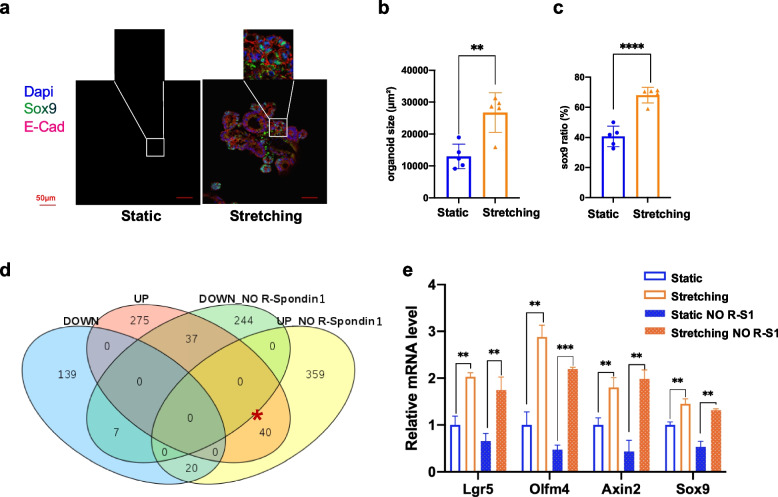
Stretching induces the Wnt/β-Catenin pathway. **a** IF staining of organoid cultivated without R-Spondin1. Organoids were stained with DAPI and immunostained for Sox9 after stretching. **b** Quantification of organoid size cultivated without R-Spondin1. **c** Quantification of Sox9+ cell ratio of organoid cultivated without R-Spondin1. **d** Venn diagram showing the overlap among up-regulated genes (red), down-regulated genes (blue), NO R-Spondin1 up-regulated genes (yellow), NO R-Spondin1 down-regulated genes (green). The significance was evaluated by Fisher’s exact test. **e** To test the expression level of Lgr5, Ascl2, Sox9, and Olfm4 using qRT-PCR. Scale bar, 50 μm

Great differences both in size and stemness were observed (Fig. 6a-c). Organoids cultured in the absence of R-Spondin1 were collected for RNA-seq and subsequent analysis. Venn diagram showed the overlap of DEGs in stretched organoids untreated or treated with R-Spondin1 (Fig. 6d). Stretched organoids treated and untreated with R-Spondin1 shared 40 common up-regulated genes, including stem cells markers and Wnt/β-catenin signaling targets, such as Lgr5, Olfm4, Axin2, Misl (Fig. 6d and Table S3). Quantitative real-time PCR was used to check the transcription level of Wnt/β-Catenin targets (e.g., Lgr5, Axin2, Sox9, and Olfm4) in the stretched and unstretched organoids. Strikingly, although the expression of various Wnt-related genes was significantly reduced after R-Spondin1 removal in static culture, there were no significant differences for organoids with or without the addition of R-Spondin1 in stretched groups (Fig. 6e). In addition, more β-catenin accumulated within crypts in stretched organoids in contrast to static ones when cultivated without R-Spondin1 (Fig. S7). These data demonstrated that a decreased expression of Wnt/β-Catenin genes resulting from loss of R-Spondin1 could be rescued by a mechanical stretch that activated the Wnt/β-Catenin signaling.

## Discussion

Here, we developed a mechanically induced dynamic intestinal organoid culture system by combining cyclic stretch with the conventional static culture model. Stretching with 8% strain at the frequency of 0.2 Hz with the sine wave shape boosted organoid growth (Fig. 2), promoting stem cell proliferation (Fig. 4 and Fig. S3) and up-regulating stemness relevant gene expression (Fig. 5). Interestingly, the effects of mechanical stretch on organoid growth were very pronounced after R-Spondin1 removed from the culture medium (Fig. 6). These experimental results potentially help us gain a better understanding of interactions between mechanical cues and biological factors during intestinal organoid generation processes. It has been widely demonstrated that the intestinal epithelium experiences comprehensive mechanical forces during gut function and organogenesis (Durel and Nerurkar 2020; Gayer and Basson 2009; Wang et al. 2019). The functional integration of mechanical forces in organoid culture provides advantages of recapitulating cyclic stretching deformation of the physical state over conventional culture, which lacks mechanical cues.

Intestinal organoids exposed to mechanical stimuli tend to generate more crypts (Fig. 3). However, the underlying mechanism still remains unclear. In vivo, Myosin II-dependent apical constriction is inevitable for initial crypt invagination (Sumigray et al. 2018). In organoids, actomyosin-driven crypt apical contraction and villus basal tension work synergistically with lumen volume reduction to drive crypt morphogenesis (Yang et al. 2021). Several studies suggest that Myosin II accumulation is mechanical stress-dependent (Fernandez-Gonzalez et al. 2009; Kee et al. 2012). In another investigation on intestinal organoids, lumen inflation stretches the epithelial monolayer and induces a stretch-associated cell state, which collectively induces crypt formation (Tallapragada et al. 2021). To this end, we speculate that stretching-induced tension within organoids might cause a stretch-responsive cell state, induce myosin re-arrangement and accumulation, and ultimately lead to crypt hyperplasia (Fig. 3).

Cell proliferation of intestinal epithelium in response to mechanical strain is validated with both the in vivo study model (Spencer et al. 2006) and the in vitro immortalized cell lines (Blair et al. 2015; Zhang et al. 2006; Zhang et al. 2003). In a study on HIOs engrafted into mice with a nitinol spring for the purpose of incorporating mechanical strain, enhanced cell proliferation was observed in stretch-applied engrafts in comparison to a sham experiment (Poling et al. 2018). In regard to our study, cyclic stretching induced the expansion of intestinal organoids. Vigorous cell proliferation, especially stem cell proliferation, was observed within the stretched organoid culture (Fig. 4, Fig. S5b, Fig. S6). We speculate that it might be owing to the stem cell activation in response to mechanical stretching that fueled cell proliferation, thus boosting the robust growth of the organoid culture.

We have testified that the Wnt/β-Catenin signaling pathway can be initiated by stretching, but it is still not clear how extracellular mechanical forces can be propagated intercellularly and produce the effects. It is well-known that Hippo-YAP/TAZ pathway is responsible for mediating mechanical signal transduction and acting as a central role in organ size control via regulation of proliferation and apoptosis (Blair et al. 2015; Dupont et al. 2011; Gregorieff et al. 2015). It might be a promising transducer to fill in the gap between the stretching stimulation and the Wnt/β-Catenin signaling activation. Additionally, a recently published literature has proposed a concept of “molecular crowding” to elegantly explain the Wnt/β-Catenin activation induced by extracellular physical/mechanical cues such as mechanical compression, osmotic pressure, stretch, matrix rigidity (Li et al. 2021). The ‘molecular crowding’ model provides a possible explanation for our experimental findings. It is likely that cyclic stretching acting on the organoids causes ‘molecular crowding’ in cell plasma membranes, thereby activating Wnt/β-Catenin by modulating the LRP6 signalosome.

## Conclusions

Altogether, we have advanced the in vitro intestinal organoid culture method by integrating cyclic stretch with the static culture system, which provides more suitable growth conditions for organoid generation. It also offers an ideal research model for understanding cross-talk between biological and mechanical factors during organoid morphogenesis. In terms of the stretch-induced activation of the Wnt/β-Catenin signaling pathway, we anticipate that the presented mechanical stretching approach can be applied to substitute growth factor R-Spondin 1 or reduce its addictive dosage for intestinal organoid culture. This model can be extended to a wide range of organoid cultures, e.g., gastric, lung, or other organoid types which are supposed to be sustained under mechanical stretch, or extended to the study of human primitive or patient-derived organoids. Our work offers a new perspective on optimizing organoid generation systems through understanding cross-talk between biotic and abiotic factors, providing potential approaches for the application of mechanical forces in organoid-based models.

## Methods

### Mice

Wild-type C57BL/6 J or heterozygous LGR5-eGFP-IRES-CreERT2 (Jackson Laboratory) were applied to isolate crypts for organoid cultivation. All breeding and experimental procedures were performed in accordance with the relevant guidelines and regulations.

### Mouse intestinal crypt isolation

Mouse intestinal crypts were isolated following the previously described protocol (Sato et al. 2009). In brief, the proximal part of the intestine was collected, opened longitudinally, washed with ice-cold PBS, scraped gently to remove villi, and chopped into 2–4 mm pieces. The sliced fragments were incubated with ice-cold PBS containing 20 mM EDTA for 30 min, then washed again with PBS 3 times, and re-suspended in an appropriate volume of PBS. Crypts were released by manually pipetting up and down several times. The supernatant was collected, passed through a 70 μm strainer, and subjected to a centrifuge at 150 g for 2 min. Finally, the supernatant was removed, leaving crypts pelleted in the bottom.

### Organoid culture and passaging

Isolated crypts were mixed thoroughly with Matrigel (50%)/Collagen (50%) (v/v), loaded to Flexcell tissue train system to form a gel strip, and topped with crypt culture medium, which was prepared from advanced DMEM/F12 (Invitrogen)) supplemented with Glutamax, B27, penicillin/streptomycin, B27 (Invitrogen), 1 μM N-acetylcysteine, and growth factors (10–50 ng/ml EGF, 500 ng/ml R-Spondin 1 and 100 ng/ml Noggin). For no R-Spondin1 incubation, R-Spondin1 was eliminated from this recipe. The medium was replenished every other day.

Passaging was performed with a 1:4 split. Gel strip embedding with organoid culture was harvested from scraping from tissue train plate, incubated with collagenase (Sigma, 1 mg/ml) for 5 min, then added 10 ml DMEM medium with 10% FBS (Fetal Bovine Serum) to inactivate collagenase. After spin and wash, culture resuspended in ~ 2 ml PBS buffer was broken by pipetting up and down several times on ice and submitted for centrifuge (200 g, 5 minutes). Pellet was mixed with an appropriate volume of the matrix, loaded to a tissue train plate to form a gel strip, and maintained for one day of static incubation before mechanical stretching.

### Mechanical stretching assay

For linear-shaped constructs, a linear ‘Trough loader’ was first placed in a loading station beneath the flexible membrane of the ‘Tissue Train’ culture plate, a 6-well plate with both silicone elastomer bottom and nylon anchor stems so that the anchor stems were aligned along the long axis of the Trough loader. The plate was assembled with a baseplate using gaskets and connected to a vacuum source (~ 90 kPa) in a steady ‘hold’ mode so that the flexible membrane was deformed and held in the space in the Trough Loader. Crypts or organoid breaking for passaging were mixed with matrix and loaded into the ‘Trough’ by sticking gel with anchor stem at both ends and maintained at 37 °C for gelation. A total of isolated ~ 300 crypts in a 200 μl matrix (composed of matrigel (50%)/Collagen (50%) (v/v)) was for each well of the plate. To apply uniaxial stretching to 3D linear construct, gel seeded ‘Tissue Train’ plate topped with the medium was transferred to Arctangel Loading station. Flexcell Software was adopted to apply a regimen of controlled wave-shape, elongation, frequency, and amplitude. Cyclic stretch was supplied through membrane distension induced by air vacuum suction at the bottom of the plate, causing the flexible plate to stretch across an Arctangle loading post. This created a uniform uniaxial strain with negligible fluid shear stress.

### RNA extraction and reverse transcription

Harvested organoid samples were dissolved in Trizol (Invitrogen Cat# 15596026) to keep in a − 80 °C freezer for a couple of weeks or to promptly perform total RNA extraction by using RNeasy Mini Kit (Qiagen) according to the manufacturer’s instructions. RNA was subjected to concentration measurement by using NanoDrop (Thermo Fisher) and cDNA synthesis with the GoScript Reverse Transcription System (Promega).

### Quantitative real time-PCR (qRT-PCR)

Real-time PCR was performed using the Roche LightCycler96 System. Primer pairs used were listed in supplementary Table 4. Expression data were normalized to the geometric mean of the housekeeping gene GAPDH to control the variability in expression levels and calculated as 2^- [(CT of indicated genes) - (CT of GAPDH)]^, where CT represented the threshold cycle for each transcript.

### RNA-Seq

RNA freshly isolated from intestinal organoids was converted into cDNA libraries using the TruSeq Illumina mRNA Library Prep. Paired-end sequencing was performed with the Illumina HiSeq2500 platform. The RNA-Seq data were uniquely mapped to the mm10 genome by TopHat v1.4.1. Expression values were assigned to gene level by Cufflinks v1.3.0. Genes with absolute log2-transformed-fold changes of more than 1.7 were regarded as differentially expressed genes, and a threshold *p*-value of less than 0.01 was used. Hierarchical clustering of log2-transformed fragments per kilobase million was generated by R. Gene set enrichment analysis was performed with GSEA v3.0 software (available from the Broad Institute).

### Paraffin section

Harvested organoids were fixed in 4% Paraformaldehyde for 3 hr., washed with PBS, and embedded with 1.5% agarose in a 1.5 ml tube. Gelated agarose block containing organoids was first placed in 10% formalin for ~ 16 hr., then shifted to dehydration by using ascending series of ethanol (EtOH). Dehydrated agarose was placed in xylene for ~ 3 hr. (change xylene every hour) and 65 °C paraffin bath overnight successively, according to a standard histological protocol, and finally embedded with paraffin. The paraffin-embedded organoid block was sectioned as thin as 4–5 μm with a microtome, floated in a 37 °C water bath containing deionized water, and mounted onto clean glass slides (coated with a tissue adhesive) so that they could be stored for at least several months or dried at 65 °C for immunostaining.

### Immunohistochemistry

Paraffin sections were routinely deparaffinized in xylene and rehydrated in decreased alcohol gradient. After a brief wash with deionized water, tissue antigen was retrieved through heating in sodium citrate buffer for 15 min, followed by incubation with 3% H_2_O_2_ for 10 min to quench the endogenous peroxidase. Following three times of PBS washing, non-specific antigen-binding sites were blocked with 10% goat serum in PBS (Gibco) overnight. The sections were then incubated with anti-Sox9 (1:5000, EMD Millipore, Cat# AB5535); anti-ki67(1:500, BD, Cat# 550609); anti-MMP-7 (1:100, Cell Signaling Technology, Cat# 3801S); anti-Olfm4 (1:500, Cell Signaling Technology, Cat# 39141S) at 4 °C overnight. After washing in PBS, the sections were incubated with biotinylated goat anti-rabbit IgG (1:1000, Vector Cat# BA-1000) or biotinylated goat anti-mouse IgG (1:1000, Vector Cat# BA-9200) for 2 hr. in room temperature. The detection was performed with a DAB detection kit (Vector Laboratories, Cat# SK-4100) and a VECTASTAIN kit (Vector Laboratories, Cat# PK-7100) according to the manufacturer’s instructions. Following counter-staining in Hematoxylin (Solarbio® LIFE SCIENCE Cat# H8070) and mounting with neutral balsam (Solarbio® LIFE SCIENCE Cat# 96949–21-2). Sections were observed using a light-field microscope (Leica DMI3000B).

### Immunofluorescence staining

Immunofluorescence staining for sectioned organoids started by dewaxing section samples in xylene, re-hydrating in a decreased alcohol gradient, retrieving antigen in boiled sodium citrate buffer, and endogenous peroxidase quenching with 3% H_2_O_2_ as described in immunohistochemistry. Following three times of PBS washing, non-specific antigen-binding sites were blocked with 10% goat serum in PBS (Gibco) overnight. The sections were then incubated with anti-Sox9 (1:1000, EMD Millipore, Cat# AB5535); anti-ki67(1:500, BD Biosciences, Cat# 550609); anti-MMP-7 (1:100, Cell Signaling Technology, Cat# 3801S); anti-YAP (1:100, Cell Signaling Technology, Cat# 14074); anti-E-Cadherin (1:500, Cell Signaling Technology, Cat# 14472); anti-β-Catenin (1:100, Cell Signaling Technology, Cat# 8480S) at 4 °C overnight. After washing in PBS, the sections were incubated with goat anti-rabbit IgG Alexa Fluor® 488 conjugate (1:1000, Cell signaling Technology Cat# 4412S) or goat anti-mouse IgG Alexa Fluor® 594 conjugate (1:1000, Cell signaling Technology Cat# 8890S) for 2 hours at room temperature. The sections were finally incubated with DAPI to stain nuclei. Sections were kept from light before they were observed with a confocal microscope (Leica SP8).

Whole-mounting of organoids for immunofluorescent staining was performed as follows. Organoids were fixed with 4% paraformaldehyde at room temperature for 2 hours. After rinsing with PBS, samples were penetrated with 0.2% Triton X-100 (Sigma-Aldrich) in PBS for 1 hr. at room temperature, and subsequently blocked in 10% goat serum in PBS (Gibco) overnight. Organoids were then successively incubated with primary antibodies and secondary antibodies, and finally stained with DAPI. Antibodies used for whole-mounting were similar to that of paraffin-sectioned samples. Organoids were pipetted onto glass slides and imaged with a confocal microscope (Leica SP8).

### Microscopy and image processing

Bright-field imaging of living organoids and organoid cross sections were performed using a Leica inverted microscope system (Leica DMI3000B) equipped with a 4×/0.10, 10×/0.22 and 20×/0.30 air objectives and TOUPCAM Touptek (Hangzhou ToupTek Photonics Co, Ltd) cameras and controlled by ToupTek ToupView (Hangzhou ToupTek Photonics Co, Ltd) software. Fluorescent imaging of organoid samples was performed using a confocal microscope (Leica SP8) equipped with 10×/0.40, 20×/0.50, and 40 × 0.3 air objectives, 405-nm, 488-nm, and 594-nm lasers and controlled by Leica Application Suite X (LAS X) software.

Cross sections of organoids along a horizontal direction were quantified in work. In brief, several random pictures were acquired from each slide using a Leica inverted microscope. The area measurement was performed by outlining each organoid using the ImageJ software. We use the “straight” tool to draw a line as long as the scale bar, and use the “set scale” function to set the distance on the picture to be the same as the scale bar. Then we use the “freehand selections” function to draw the outline of each organoid, and finally, we obtain the area of the organoid automatically through the “measurement” function. Only complete organoids were selected for the measurement.

### Statistical analysis

Data were expressed as means ± SD of at least three independent experiments. Statistical analyses were performed using GraphPad Prism software (Graphpad, La Jolla, CA). Two-tailed unpaired Student’s t-tests and a one-way ANOVA were used for comparison between two groups and three or more groups, respectively. *P* values < 0.05 were considered statistically significant. Bar graphs represent mean ± SEM.

## Supplementary Information

**Additional file 1: Fig. S1.** Organoid culture matrix. The up row of this picture exhibits gel stripes formed with different components. The left is pure collagen, with pure Matrigel on the right and Matrigel (50%)/Collagen (50%) (v/v) in the middle. Crypts cannot generate into organoids in pure collagen, whereas both pure Matrigel and Matrigel-Collagen mixture can support organoid grow (bottom row). Scale bar, Scale bar, 50 μm. **Fig. S2.** Effect of strain timing window and frequency of force loading on organoid morphogenesis. **a** Imaging of 10% strain at the frequency of 0.2 Hz started stretching on days 0, 1, 2, 3, 4 after crypt isolation. **b** Imaging of 8% strain applied during Day3–6 at variable frequencies ranging from 0.02–5 Hz. Scale bar, 100 μm. **Fig. S3.** Mechanical stretching induces cell proliferation. **a** Imaging and quantification of IHC staining for Ki67 in organoid cultured with 8% cyclic stretching (right panel) or under static condition (left panel) respectively. **b** Imaging and quantification of IHC staining for Mmp7 in organoid cultured with 8% cyclic stretching (right panel) or under static condition (left panel) respectively. **c** Imaging and quantification of IHC staining for Sox9 in organoid cultured with 8% cyclic stretching (right panel) or under static condition (left panel) respectively. *n* = 6 organoids derived from three independent experiments for each condition. Student’s t-test: ***P < 0.001. **P < 0.01. *P < 0.05. Scale bars, 50 μm. **Fig. S4.** Whole Mount Immunostaining for Olfm4. Use excitation wavelength 594 nm for Actin-Red, 488 nm for Olfm4-Green, 405 nm for Hoechst. **Fig. S5.** Bioinformatics analysis. **a** Volcano plots for sequencing analysis. Purple dots represent up-regulated genes, yellow dots represent down-regulated genes, and green dots stand for none differential expressing genes. **b** KEGG pathway analysis. **Fig. S6.** GO analysis. Red bars show up-regulated numbers of genes, while greens bars represent down-regulated numbers of genes. **Fig. S7.** β-Catenin immunostaining for organoids cultivated without R-Spondin1. a Paraffin section immunostaining for Olfm4. b whole mount immunostaining for Olfm4.

**Additional file 2: Supplementary Video 1.**

**Additional file 3: Supplementary Video 2.**

**Additional file 4: Supplementary Video 3.**

**Additional file 5: Supplementary Table 1.** 384 ISC Signature Genes.

**Additional file 6: Supplementary Table 2.** Overlap 69.

**Additional file 7: Supplementary Table 3.** Overlap40.

**Additional file 8: Supplementary Table 4.** Primer list.

## Data Availability

The datasets used and/or analysed during the current study are available from the corresponding author on reasonable request.

## Abbreviations

MIO: Mouse Intestinal Organoid
GSEA: Gene set enrichment analysis
ECM: Extracellular matrix
NOD-SCID: Nonobese diabetic/severe combined immunodeficiency
IHC: Immunohistochemical
IF: Immunofluorescence
ISC: Intestinal stem cell
DEGs: Differentially expressed genes
FDR: False discovery rate
