# Supplementary material for: Mechanical stretching boosts expansion and regeneration of intestinal organoids through fueling stem cell self-renewal
Source: Cell Regen. 2022 Nov 2;11:39. doi: 10.1186/s13619-022-00137-4 (PMC9626719; doi:10.1186/s13619-022-00137-4)
Supplement: Supplementary file 1 — Additional file 1: Fig. S1. Organoid culture matrix. The up row of this picture exhibits gel stripes formed with different components. The left is pure collagen, with pure Matrigel on the right and Matrigel (50%)/Collagen (50%) (v/v) in the middle. Crypts cannot generate into organoids in pure collagen, whereas both pure Matrigel and Matrigel-Collagen mixture can support organoid grow (bottom row). Scale bar, Scale bar, 50 μm. Fig. S2. Effect of strain timing window and frequency of force loading on organoid morphogenesis. a Imaging of 10% strain at the frequency of 0.2 Hz started stretching on days 0, 1, 2, 3, 4 after crypt isolation. b Imaging of 8% strain applied during Day3–6 at variable frequencies ranging from 0.02–5 Hz. Scale bar, 100 μm. Fig. S3. Mechanical stretching induces cell proliferation. a Imaging and quantification of IHC staining for Ki67 in organoid cultured with 8% cyclic stretching (right panel) or under static condition (left panel) respectively. b Imaging and quantification of IHC staining for Mmp7 in organoid cultured with 8% cyclic stretching (right panel) or under static condition (left panel) respectively. c Imaging and quantification of IHC staining for Sox9 in organoid cultured with 8% cyclic stretching (right panel) or under static condition (left panel) respectively. n = 6 organoids derived from three independent experiments for each condition. Student’s t-test: ***P < 0.001. **P < 0.01. *P < 0.05. Scale bars, 50 μm. Fig. S4. Whole Mount Immunostaining for Olfm4. Use excitation wavelength 594 nm for Actin-Red, 488 nm for Olfm4-Green, 405 nm for Hoechst. Fig. S5. Bioinformatics analysis. a Volcano plots for sequencing analysis. Purple dots represent up-regulated genes, yellow dots represent down-regulated genes, and green dots stand for none differential expressing genes. b KEGG pathway analysis. Fig. S6. GO analysis. Red bars show up-regulated numbers of genes, while greens bars represent down-regulated numbers of genes. Fig. S7. β-Cat [file 13619_2022_137_MOESM1_ESM.docx]

**Supplementary figures**

**Mechanical Stretching Boosts Expansion and Regeneration of Intestinal Organoids through fueling stem cell self-renewal**

*Fanlu Meng^§^, Congcong Shen^§^, Li Yang, Chao Ni, Jianyong Huang, Kaijun Lin, Zanxia Cao, Shicai Xu, Wanling Cui, Xiaoxin Wang, Bailing Zhou, Chunyang Xiong^*^, Jihua Wang^*^ and Bing Zhao^*^*


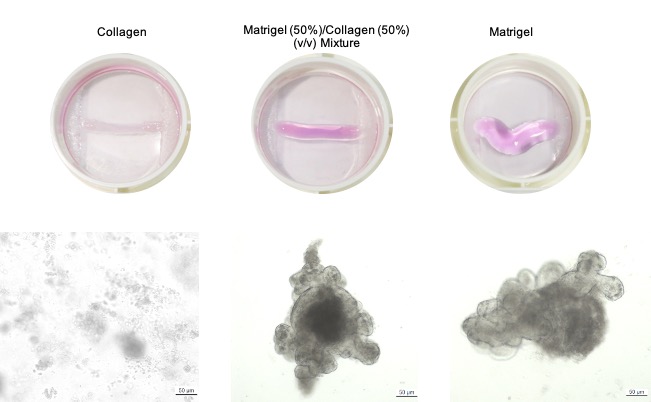


**Figure S1.** Organoid culture matrix. The up row of this picture exhibits gel stripes formed with different components. The left is pure collagen, with pure Matrigel on the right and Matrigel (50%)/Collagen (50%) (v/v) in the middle. Crypts cannot generate into organoids in pure collagen, whereas both pure Matrigel and Matrigel-Collagen mixture can support organoid grow (bottom row). Scale bar, Scale bar, 50 μm.


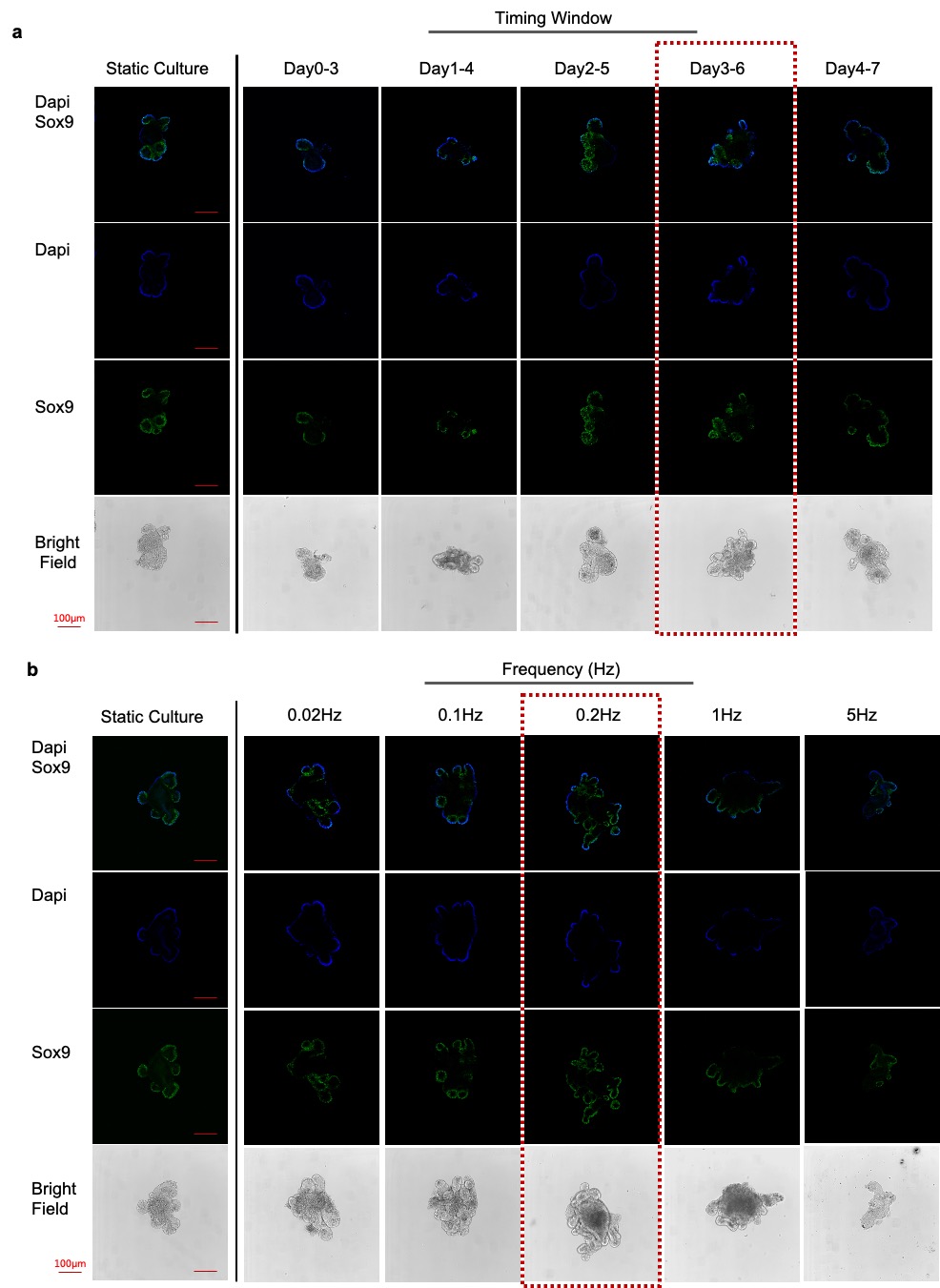


**Figure S2.** Effect of strain timing window and frequency of force loading on organoid morphogenesis. **a** Imaging of 10% strain at the frequency of 0.2 Hz started stretching on days 0, 1, 2, 3, 4 after crypt isolation. **b** Imaging of 8% strain applied during Day3-6 at variable frequencies ranging from 0.02-5 Hz. Scale bar, 100 μm.

**
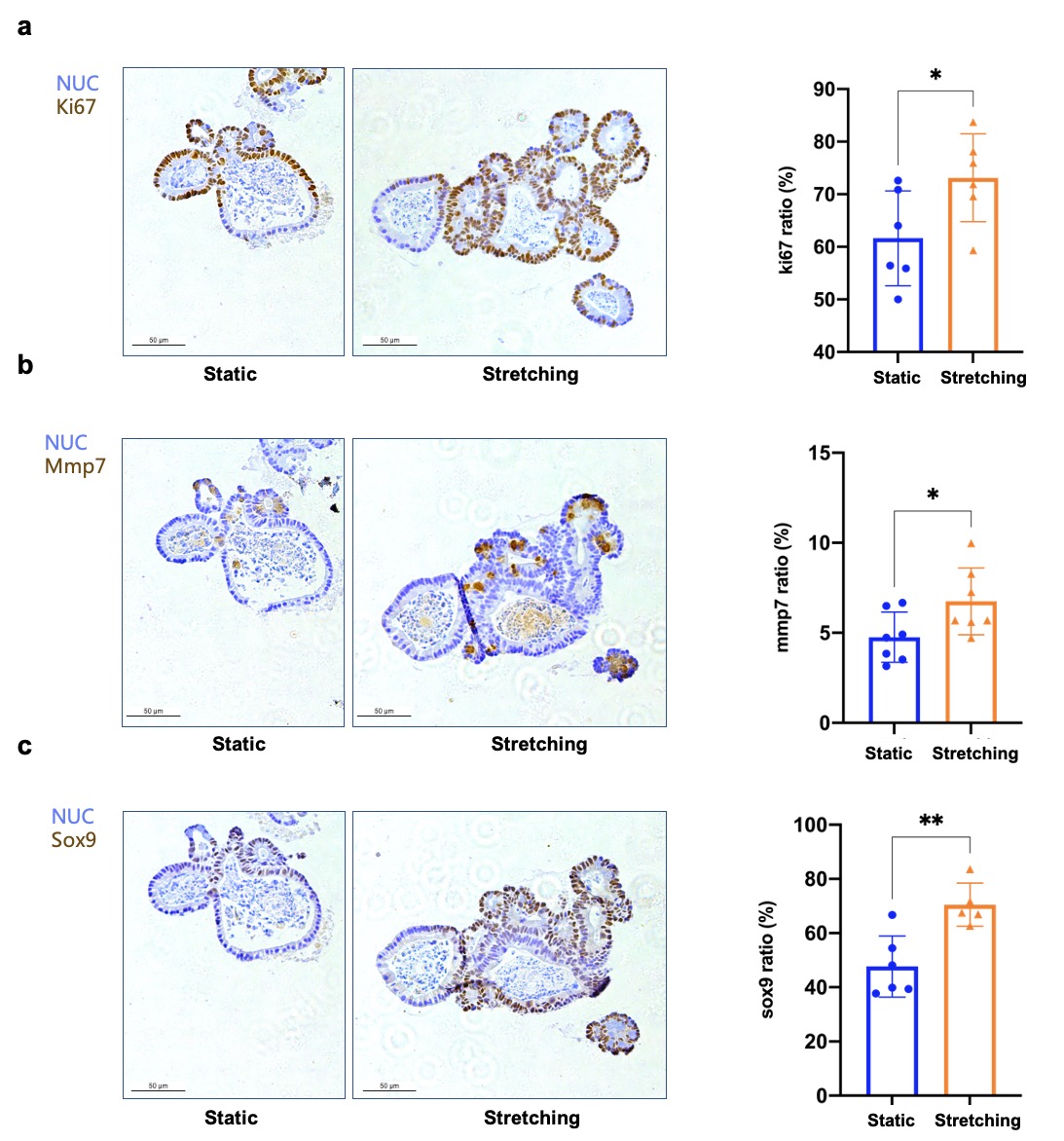
Figure S3.** Mechanical stretching induces cell proliferation. **a** Imaging and quantification of IHC staining for Ki67 in organoid cultured with 8% cyclic stretching (right panel) or under static condition (left panel) respectively. **b** Imaging and quantification of IHC staining for Mmp7 in organoid cultured with 8% cyclic stretching (right panel) or under static condition (left panel) respectively. **c** Imaging and quantification of IHC staining for Sox9 in organoid cultured with 8% cyclic stretching (right panel) or under static condition (left panel) respectively. n=6 organoids derived from three independent experiments for each condition. Student’s t-test: ***P < 0.001. **P < 0.01. *P < 0.05. Scale bars, 50μm.

**
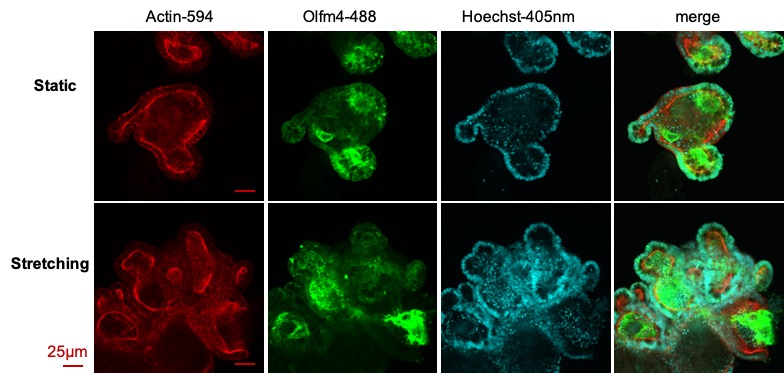
Figure S4.** Whole Mount Immunostaining for Olfm4. Use excitation wavelength 594nm for Actin-Red, 488nm for Olfm4-Green, 405nm for Hoechst.

**
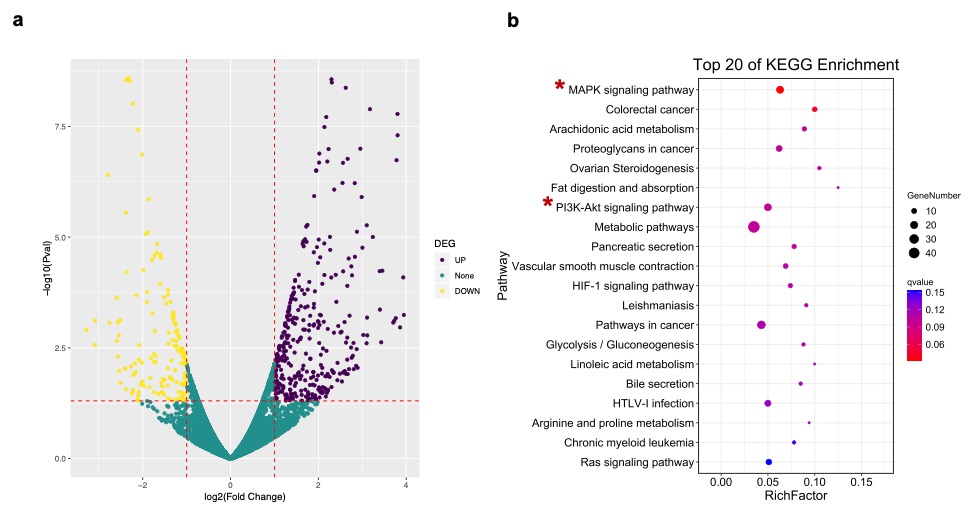
Figure S5.** Bioinformatics analysis. **a** Volcano plots for sequencing analysis. Purple dots represent up-regulated genes, yellow dots represent down-regulated genes, and green dots stand for none differential expressing genes. **b** KEGG pathway analysis.

**
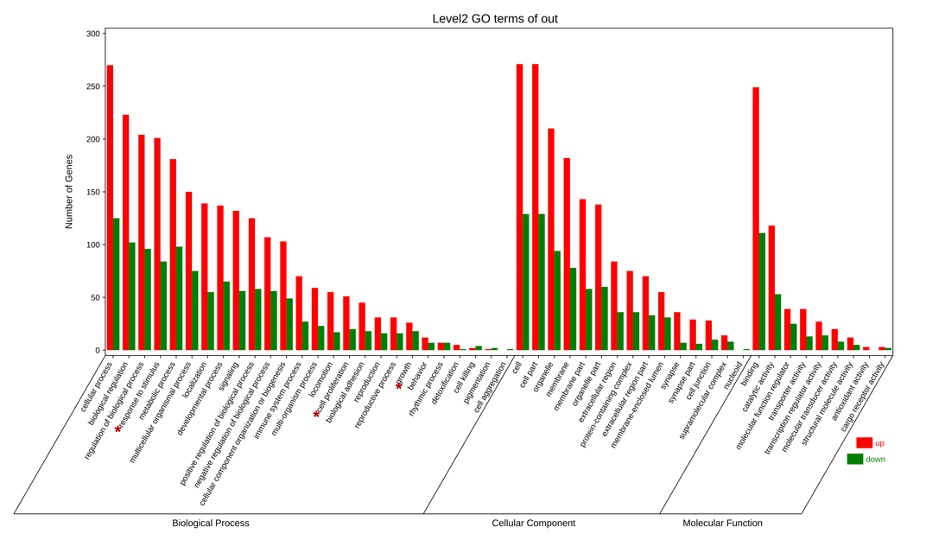
Figure S6.** GO analysis. Red bars show up-regulated numbers of genes, while greens bars represent down-regulated numbers of genes.


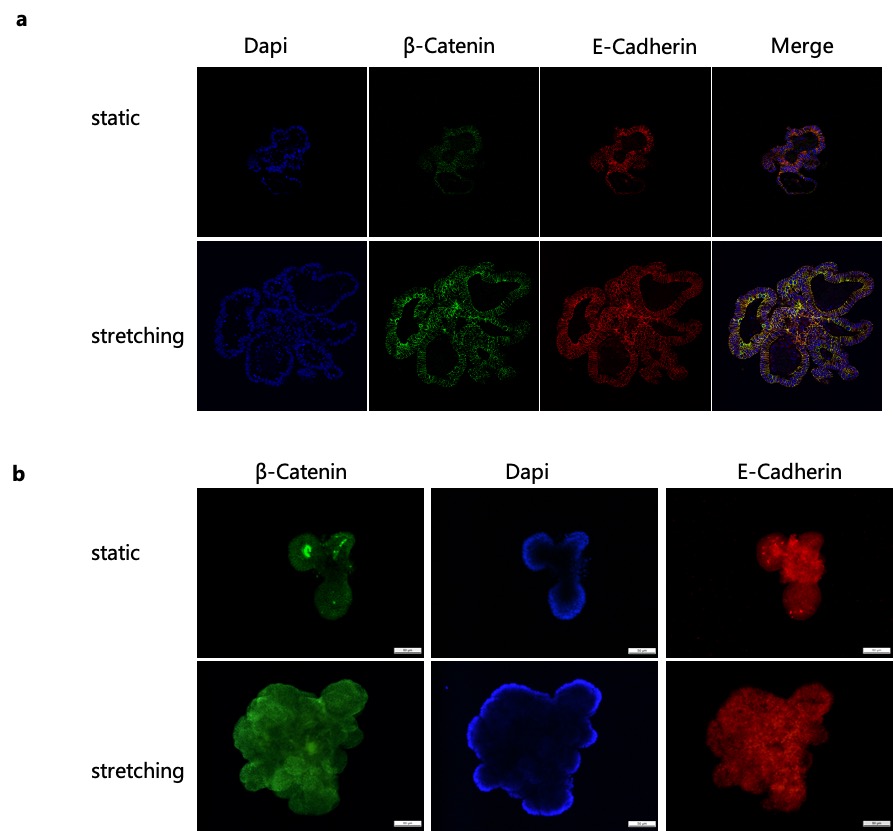


**Figure S7.** β-Catenin immunostaining for organoids cultivated without R-Spondin1. **a** Paraffin section immunostaining for Olfm4. **b** whole mount immunostaining for Olfm4.
